# Cumulative semantic cost without successful naming

**DOI:** 10.3758/s13421-021-01172-3

**Published:** 2021-03-29

**Authors:** Eduardo Navarrete, Silvia Benavides-Varela, Riccardina Lorusso, Barbara Arfè

**Affiliations:** Dipartimento di Psicologia dello Sviluppo e della Socializzazione, Università di Padova, Via Venezia 8, 35131 Padova, Italy

**Keywords:** Interference/inhibition in memory retrieval, Psycholinguistics, Semantic priming, Word production, Lexical processing, Picture naming in children

## Abstract

Accessing semantic information has negative consequences for successive recovering attempts of similar information. For instance, in the course of picture-naming tasks, the time required to name an object is determined by the total number of items from the same category that have already been named; naming latencies increase proportionally to the total number of semantically related words named previously. This phenomenon is called cumulative semantic cost (or interference). Two picture-naming experiments with children (4–11 years old, 229 participants) investigate whether having successfully named the previous within-category items is a necessary condition for the cumulative semantic cost to appear. We anticipated that younger children would have a larger rate of nonresponses compared with older children, reflecting the fact that younger children have not yet consolidated many lexical representations. Our results confirmed this prediction. Critically, we also observed that cumulative semantic cost was independent of having successfully retrieved previous within-category lexical items. Furthermore, picture trials for which the previous within-category item elicited a nonresponse showed the same amount of cost as those picture trials for which the previous within-category item elicited a correct naming event. Our findings indicate that it is the attempt to retrieve a lexical unit, and not the successful retrieval of a specific lexical unit, that causes semantic cost in picture naming. This cost can be explained by a mechanism of weakening the semantic-to-lexical mappings of semantic coordinate words. The findings are also discussed in the context of retrieval-induced forgetting effects in memory recall research.

In naming an object, before initiating lexical access, speakers retrieve the semantic representation corresponding to the picture of the object. Researchers have captured the notion of spreading activation to describe how activation flows through the semantic and the lexical systems. It is generally assumed that any activated concept spreads activity to a cohort of related concepts, which in turn spread activation to the lexical system (e.g., Dell, 1986; Rapp & Goldrick, 2000). Consequently, in the course of lexical access, multiple lexical representations are activated—that is, the target along with semantically related items. A second universally agreed-upon conviction is that the speech production system retains some amount of activation for a certain period of time. Evidence from the picture-naming task seems congruent with these assumptions. For instance, a semantic priming effect similar to that observed in the word-recognition paradigm (see, for instance, McRae & Boisvert, 1998) occurs in picture-naming tasks: naming latencies in a given trial (*n*) are faster if a related (semantic coordinate) item has been named in the immediately preceding trial (*n* − 1), in comparison with when an unrelated item is named (Huttenlocher & Kubicek, 1983; Lupker, 1988). Semantic priming finds a natural explanation in terms of spreading activation between semantically related representations.

Interestingly, the semantic priming reverses if an unrelated picture is presented between the two related pictures. That is, naming latencies in trial *n* are slower when a related target has been named in trial *n −* 2 (e.g., Tree & Hirsh, 2003; Wheeldon & Monsell, 1994). Semantic interference seems to be a long-lasting phenomenon, in which interference is propagated across many (unrelated) intervening trials or for several seconds; interference is also reliable when several unrelated pictures are presented between the two related trials, for instance at lags larger than *n* − 2. Furthermore, the interference increases for each item proportionally to the total number of items from the same semantic category that have already been named (Brown, 1981; Howard et al., 2006). This kind of long-lasting and cumulative semantic cost has recently attracted much interest from models of lexical access in word production (see, e.g., Belke & Stielow, 2013).^1^

The first account used to explain cumulative semantic cost was proposed by Howard et al. (2006). According to them, the phenomenon would arise as a consequence of the convergence of three properties. One is priming, which refers to the assumption that when a representation is activated, it will retain that activation for a certain amount of time. A second property is shared activation, which refers to the assumption that activation spreads to semantically related words when naming a target word. In this manner, when producing, for instance, the word *car,* the related lexical entries for the words *truck* and *van* will also become activated, and this activation would be retained for a certain period. As described in the first paragraph, there exists general agreement regarding these two first properties. However, the third property proposed by Howard and colleagues has been the subject of theoretical disagreement. This property refers to the assumption that lexical retrieval (i.e., selection) is a competitive process, meaning that the time required to retrieve a word depends on the levels of activation of other activated but nontarget words. In the specific computational model developed by Howard and colleagues, competition is implemented by lateral inhibition between lexical candidates—that is, each lexical unit (i.e., lemma in their model) inhibits other lexical units in proportion to its own activation level. Thus, according to the lexical competition property, when an item is named (*car*), the semantic-to-lexical connections for that item are strengthened and retain activation, becoming a stronger lexical competitor when a semantic coordinate item has to be named (*truck*) later on.

A second approach aims to explain that the cumulative semantic cost implements an incremental learning mechanism by which semantic-to-lexical connection weights are adjusted after each naming event. This approach was developed by Oppenheim et al. (2010) and states that naming a picture strengthens connections between the semantic and lexical representations of that word (e.g., *car*) and, at the same time, weakens the connections between the semantic and the lexical representations corresponding to semantic coordinate words (e.g., *truck*, *van*). When one of these words has to be named later on, latencies will be slower because of the weakened semantic-to-lexical connections. According to this approach, the semantic cost is generated during the lexicalization process to produce an item (*car*), even if it is only observed some trials later during the lexicalization of a within-category item (*truck*).

These two approaches diverge on the attribution of cumulative semantic cost in picture naming. While the competitive explanation assumes that the cost is due to the strengthening of lexical competitors that hampers the lexical selection of the upcoming targets (Howard et al. 2006); the weakening account attributes the effect to an inhibitory process on the semantic-to-lexical connections corresponding to the upcoming targets (Oppenheim et al., 2010). Oppenheim and collaborators (2010) provided the first test of these two approaches. In a series of simulations, they tested a computational model without a competitive lexical selection rule but with a weakening (inhibitory) mechanism between the semantic and lexical representations of semantic coordinate words. Simulations 5 and 6 of their study reported semantic costs under these circumstances, suggesting that lexical competition would not be a necessary condition for the cumulative semantic cost to emerge and that the weakening (inhibitory) mechanism is sufficient to explain it.

Other empirical studies exploring the boundaries of the cumulative semantic cost are relevant for our purposes here. Research by Navarrete et al. (2010, 2016) has reported that the cumulative semantic cost is ascribed to those circumstances in which participants are required to ‘actively retrieve’ category-exemplars from their mental lexicon. In a series of experiments, German and Italian participants were exposed to a sequence of intermingled words and pictures and instructed to name them with the corresponding gender-marked determiner. Grammatical gender is a syntactic feature of nouns and cannot be derived from conceptual properties; therefore, even though a printed word is presented, the lexical representation corresponding to the word must be retrieved to access its grammatical gender (e.g., Damian et al., 2001; Jescheniak et al., 2001). In these experiments, interference was obtained for both word and picture targets, but only when the preceding within-category items were pictures, and not when they were words (but see Belke, 2013). Navarrete and colleagues concluded that picture naming entails adjustments on the semantic-to-lexical connections for semantic coordinates of the target picture that will affect the time required to retrieve lexical representations on subsequent within-category trials, irrespective of their format (i.e., picture or word). By contrast, naming a word does not entail weakening modifications on the semantic-to-lexical mappings, and therefore, naming latency to retrieve lexical representations on subsequent within-category trials is unaffected (again, irrespective of its format, i.e., picture or word). These results seem to suggest that ‘lexical retrieval’ per se does not cause cost; that is, cumulative semantic cost would be not ascribed to the retrieval of the lexical unit.

Here, we adopt a different strategy to test both the weakening and the competition accounts. In two studies, we separately test whether lexical retrieval success is a necessary condition for cumulative semantic cost to emerge. Specifically, we examined the cumulative semantic cost in a population with a high rate of unsuccessful lexical retrieval attempts, as is the case of preschool and primary school children.

## The present research

Unlike adults, children have semantic representations of objects for which they may lack in production, a corresponding label. Indeed, in their second semester of life, before infants can talk, they start understanding the meanings of many common nouns (Bergelson & Swingley, 2012) and represent the semantic relations between these words (Bergelson & Aslin, 2017). Moreover, during the second year of life, infants begin teasing apart distinct kinds of names (e.g., nouns, adjectives) and their relation to distinct kinds of concepts (e.g., object categories, properties; Ferguson & Waxman, 2017). This reflects an early organization of the knowledge of words, including words that children may be able to utter and efficiently use/retrieve only later, throughout childhood.

In the two picture-naming studies presented here, with preschool and primary school children, we aimed at evaluating (a) the developmental trend of nonresponses, (b) the developmental trend of semantic costs and, most critically, and (c) the interaction between the two. Because vocabulary continues to increase during childhood (Riva et al., 2000; Song et al., 2015), we expected higher nonresponse rates for younger children in comparison with older children, reflecting a smaller vocabulary size for the younger group. Moreover, we predicted the presence of cumulative semantic cost—specifically, that naming latencies will grow linearly as a function of the number of previously named pictures in the same category, in agreement with recent evidence with school-age children (Charest & Baird, 2020). Under the hypothesis that the cost arises as a consequence of the weakening of the semantic-to-lexical mappings, the cumulative semantic cost may be independent of the successful naming of the target word. That is, the weakening account does not predict an interaction between number of no-responses and cumulative cost. According to this, the same amount of cumulative cost is expected irrespective of age; naming latencies in younger children (with a smaller vocabulary) would have a similar amount of linear increase as naming latencies in older children (with a larger vocabulary). By contrast, under the assumption that cumulative semantic cost depends on the level of activation of the lexical competitors, a null cost is expected in those circumstances in which the lexical competitors are not retrieved because they have not yet consolidated in the lexical system of the speaker. That is, the competition account predicts an interaction between the number of no-responses and cumulative cost. As a consequence, an interaction between cumulative cost and age is expected, with naming latencies in younger children having less amount of linear increase.

## Method

Two studies are presented. In the first study, we analyzed data previously collected in a normative naming study in Italian children (Lorusso et al., 2021). In the second study, we replicated the main findings of Study 1 with a new population of children using an experimental approach, with a controlled and factorial design. A total of 190 and 39 children were included in Study 1 and Study 2, respectively. The number of participants satisfied the required sample size based on a statistical power analysis (G*Power 3.1; Faul et al., 2007). Statistical power analysis was based on data from Charest (2017). In that study, 17 children were tested in a slightly different picture-naming task, the semantic blocking naming. We estimated the correlation index between the semantically related and the semantically unrelated conditions, *r* = .6. With alpha = .05 and power = 0.95, the anticipated sample size required to obtain a significant semantic interference effect in picture naming is *n* = 37.^2^ All data are made available under the following OSF repository (https://osf.io/k4xzr/). The studies were not preregistered.

### Study 1

#### Participants

A total of 190 children, all native Italian speakers, took part in Study 1. Data came from the normative study conducted by Lorusso et al. (2021) aimed at describing the developmental trajectory of Italian vocabulary in typically developing children. Children were recruited from different schools located in three regions of Italy: Veneto (north-west), Marche (east-center), and Puglia (south-east). Children came from seven different school levels and were between the ages of 4;4 and 11;1 (*M*_age_ = 7;9). See Table 1 for the distribution of children per school level. Children were tested individually in a quiet room at their schools. Children in both this and Study 2 had normal vision and did not present developmental disabilities or suspected language or learning delays.

**Table 1:** Demographic characteristics of the sample in Study 1: Age (years; months), age range in months, and the number of male/female and % of nonresponses in the picture-naming tasks at each school level

	Age	Age range in months	*N* (males)	% of nonresponse
Preschool (2nd)	4;9	53–63	17 (9)	12.5
Preschool (3rd)	5;9	62–77	29 (14)	10.3
Primary School (1st)	6;7	72–88	30 (12)	9.3
Primary School (2nd)	7;8	87–100	28 (16)	6.0
Primary School (3rd)	8;7	98–111	29 (12)	5.1
Primary School (4th)	9;7	109–123	28 (12)	4.8
Primary School (5th)	10;7	111–134	29 (14)	3.2

#### Materials

The experimental pool was composed of 79 pictures that can be classified as belonging to one of 10 semantic categories. Four pictures did not belong to clearly defined categories and were excluded from the analysis. The number of exemplars per category varied from 3 to 11 (see Appendix 1). Analyses were performed on 1 to 7 within-category orders only. This was done to avoid spurious effects of few items and to increase the possibility of detecting cumulative semantic costs (e.g., the category animal is the only category that would contribute to order positions 10th and 11th). In sum, each participant contributed with 62 experimental critical data points.

#### Procedure

Children were seated in front of a computer screen, wearing a headset microphone, and were asked to name the pictures as fast and as accurately as possible using a single word. In each trial, a fixation cross was shown in the center of the screen for 1,000 ms and was followed by the target pictures presented for 5,000 ms or until the participant’s response. The next trial started after 1,500 ms. Pictures were presented randomly and within-category items were separated by at least one intervening item from another semantic category. Stimulus presentation and response times were controlled by the program Catalogation Time, CAT (www.kinopsys.com).

#### Analysis

Microphone failures (28 trials, 0.23%) were discarded. On 11,752 data points, we first explored the developmental trend of nonresponse rates through the different school levels using a generalized linear mixed model analysis (GLMM).

Moreover, we analysed reaction times (RTs) in order to evaluate the developmental trend of semantic costs. Trials that did not elicit verbal responses (i.e., nonresponses, 6.5%) and those with the production of erroneous utterances (13.6%) were excluded in this analysis. RTs outliers (2.9%) were removed using Van Selst and Jolicoeur, 1994) procedure. As the RTs data were not normally distributed, we used the Box–Cox test (Box & Cox, 1964), using the function boxcox in the package MASS (Venables & Ripley, 2002) to estimate the most appropriate transformation for the RTs to reduce skewness and approximate a normal distribution. RTs were then analyzed using linear mixed model analysis (LMM) on 9,052 data points. The predictors ordinal position within category (1 to 7 orders within category) and age (in months) were entered as fixed effects in the statistical model. The interaction between ordinal position within category and age was also explored.

In a further analysis, we directly tested the interaction between cumulative semantic cost and nonresponse rates. Thus, we took into account whether the previous within-category item elicited a successful retrieval (i.e., correct naming response) or a nonresponse. Previous within-category performance (successful, nonresponse), ordinal position within category, and age were entered as fixed effects in the statistical model. The interaction between previous within-category performance and ordinal position within category was also explored. The first trial from each category was excluded from this second type of analysis, as no previous within-category item is presented for those trials; the analysis was conducted on 6,645 data points. Finally, since school level may be more correlated with vocabulary size, we performed the same series of analyses with school level (1 to 7 school levels) instead of age as predictor.

Analyses were performed using the library lme4 (Bates, Maechler, et al., 2015b) on the software R (R Core Team, 2020). Following Bates, Kliegl, Vasishth, et al.’s (2015a) suggestion against a maximal random effects approach, for the random effects part of the models we considered three random factors: participants, items, and semantic categories (see also for the same approach Runnqvist et al., 2012; Schnur, 2014).

Székely et al. (2003) reported that pictures named early in the sequence of a picture-naming task tended to be named faster than pictures named later in the sequence. That is, participants become fatigued. To rule out any fatigue effect in the context of cumulative semantic interference is of critical relevance because, by definition, later ordinal position within-category items are always named later in the experimental sequence. We controlled for this phenomenon by including the predictor “trial” in the models.

## Results

**Fig. 1:**
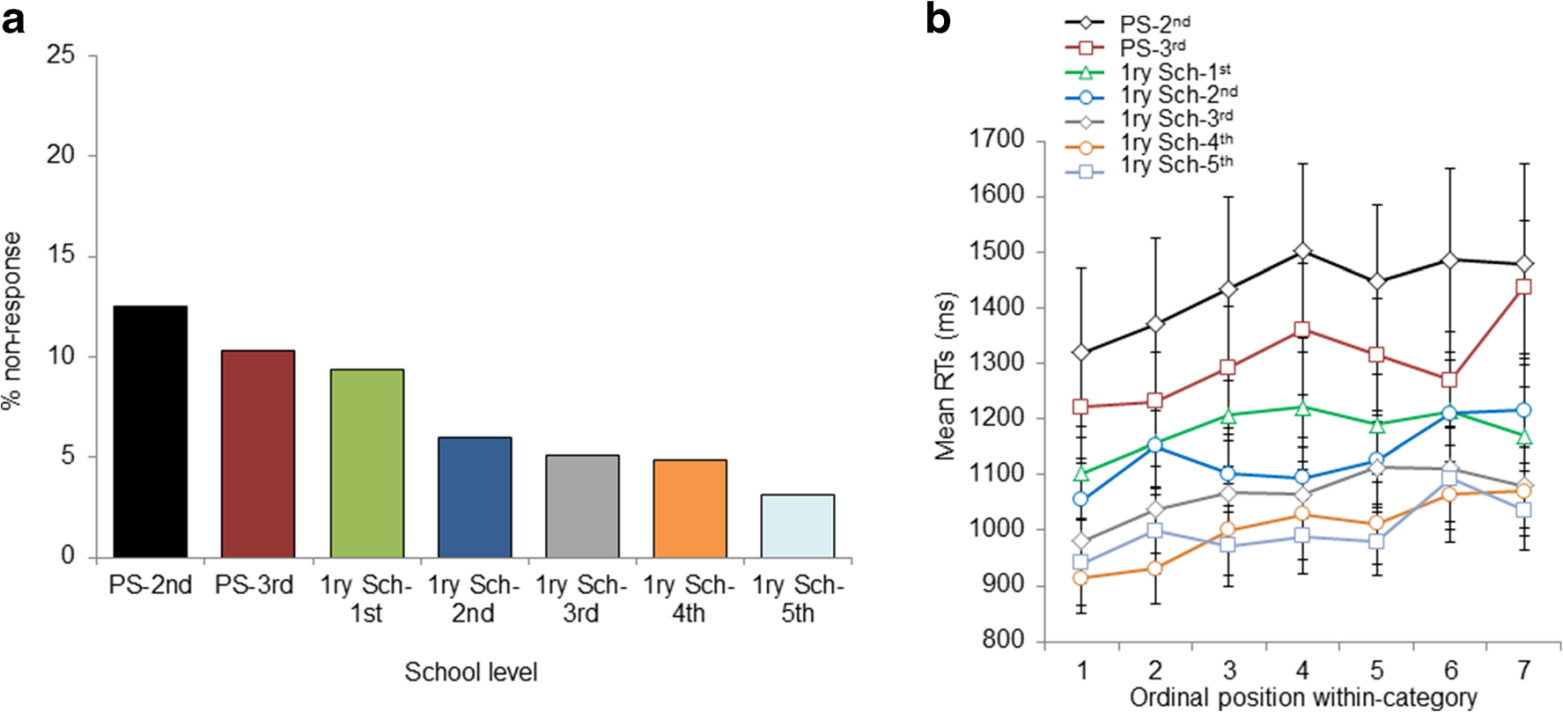
Study 1. **a** Developmental trend of nonresponses: Percentage of nonresponses in each School-level. **b** Cumulative semantic costs: Mean naming latencies by ordinal position within-category in each school level. As can be seen, naming latencies increase with ordinal position within category in all the school levels. All error bars are standard error of the mean. PS = preschool; 1ry Sch = primary school

The GLMM analysis evaluating the developmental trend of nonresponses revealed that nonresponses were more frequent in younger children than in older children, χ^2^(6) = 130.82, *p* < .001 (analysis on the factor school level), χ^2^(6) = 129.17, *p* < .001 (see Table 1 and Fig. 1).

The LMM analysis evaluating the developmental trend of cumulative semantic costs showed a significant main effect of trial, χ^2^(1) = 11.08, *p* < .001, indicating that RTs increased with trial. The main effects of ordinal position within category, χ^2^(1) = 16.77, *p* < .001, and age, χ^2^(1) = 150.19, *p* < .001, were also significant. The critical Ordinal Position Within Category × Age interaction was not significant, χ^2^(1) = 1.62, *p* = .202. The same pattern of results emerged with the factor school level: significant main effects of trial, χ^2^(1) = 11.09, *p* < .001, order, χ^2^(1) = 16.73, *p* < .001, and school level, χ^2^(1) = 155.37, *p* < .001, but no effects of the critical Ordinal Position Within Category × School Level interaction, χ^2^(1) = 2.29, *p* = .129 (see Appendix 2a and 2b for regression coefficients and standard errors). As can be seen in Fig. 1, RTs increased for each subsequent within-category item, while overall RTs were slower for younger children (earlier school level). For the sake of clarity, we plot the school-level factor in Fig. 1 instead of the factor of age.

In the analysis directly evaluating the interaction between cumulative semantic cost and nonresponse rates, we observed that RTs did not differ as a function of whether the previous within-category item was successfully retrieved or was a nonresponse trial, χ^2^(1) = .22, *p* = .638. The effects of trial, χ^2^(1) = 5.31, *p* = .021, and ordinal position within category, χ^2^(1) = 7.11, *p* = .007, were significant. The critical Previous Within Category × Ordinal Position Within Category interaction was not significant, χ^2^(1) = 0.64, *p* = .420 (see Appendix 2c for regression coefficients and standard errors).

Before discussing these results and drawing any conclusions, we will present Study 2, which aimed at replicating the findings of Study 1 (derived from previously collected data in a normative study) with a different group of children and using an experimental approach. Some methodological changes were introduced. First, it has been demonstrated that photographs increase name agreement and correct response rates in comparison to black-and-white drawings in both adults (Salmon et al., 2014) and children (Martínez & Matute, 2019). Thus, to reduce stimulus-specific effects, color photographs were used in Study 2. Second, the vocabulary of the participant was also assessed by a standardized test. Participants completed the Italian version of the Peabody Picture Vocabulary Test–Revised (PVVT-R; Stella et al., 2000) after the main picture-naming task. The PVVT-R is a norm-referenced test of receptive vocabulary breadth, widely used in developmental research to estimate vocabulary growth and discriminate between children with typical and atypical development (Rice & Hoffman, 2015). In line with Study 1, we expected a main effect of vocabulary breadth on children’s accuracy in picture naming, but no significant interaction between vocabulary scores and ordinal position within category. Third, we adopted a more controlled and factorial design. In Study 2, each category contains the same number of exemplars, and these are separated by the same number of unrelated items.

### Study 2

#### Participants

Participants were 39 children, all native Italian speakers recruited from one school located in Veneto. Children were between the ages of 4;1 and 8;2 years (*M*_age_ = 6;2). See Table 2.

**Table 2:** Demographic characteristics of the sample in Study 2: Age (years; months), age range in months, and the number of male/female, mean and standard deviant on the Peabody Picture Vocabulary Test–Revised (raw scores), and % of nonresponses in the picture-naming tasks at each school level

	Age	Age range in months	*N* (males)	Peabody Test–mean raw scores (*SD*)	% of no response
Preschool (2nd)	4;6	49–61	6 (1)	78.3 (4.5)	21.0
Preschool (3rd)	5;7	56–76	16 (9)	84.6 (7.3)	13.4
Primary school (1st)	6;7	78–85	10 (4)	87.7 (10)	11.8
Primary School (2nd)	7;9	90–99	7 (4)	108 (5.5)	9.8

#### Materials and design

One hundred and sixty-four color photographs were selected. Experimental items consisted of 96 pictures belonging to 16 different semantic categories, with six exemplars in each category. The rest of the photographs were filler items, and none of them belonged to any of the categories of the experimental items (see Appendix 3). The set of photographs were selected from Howard et al. (2006) experimental data set and from internet. The photographs were randomly inserted into a sequence with the following constraints. Pictures from each category were separated by lags of 3, 4, 5, 6, or 7 intervening items. Filler items and the order of the categories in the sequence were randomly assigned. This process was repeated nine times following the same constraints and structure, resulting in 10 experimental sequences. There was a self-paced break of a few seconds in the middle of the sequence to reduce the fatigue of the children. The first three items at the beginning of the sequence and after the break were filler items. All six within-category items were presented before or after the pause. Each participant received one experimental sequence, and each experimental sequence was used a minimum of 3 times across all the participants.

#### Procedure

The same procedure as in Study 1 was used, with the following exceptions. In each trial, a fixation cross was shown in the center of the screen for 600, 700, 800, or 900 ms and was followed by a blank interval lasting 400 ms. Stimulus presentation, response times, and response recording were controlled by the program DMDX (Forster & Forster, 2003).

After the picture-naming task, participants were exposed to the PVVT-R (Stella et al., 2000). In this test, the child is asked to choose from a selection of four pictures the one that represents the word pronounced by the examiner. Raw scores can be converted in age-norm referenced standard scores.

#### Analysis

The same analyses as in Study 1 were performed, with the difference that the interaction between the PVVT-R score and the ordinal position within category was also explored. PPVT-R raw scores were used in the analyses to account for developmental differences in vocabulary breadth. Preliminary *t*-test statistics confirmed that the four age (i.e., school level) groups differed significantly for vocabulary breadth, *p*s < .001. Standard scores were used to ascertain that all children performed within their age norms (standard scores equivalent >.85). Nonresponses (11.8%) and erroneous utterances (7.9%) were removed from the RT analysis. RT outliers (2.3%) were also removed. RTs analyses were performed on 2,923 in the first and 2,060 in the second level of analysis.

## Results

**Fig. 2:**
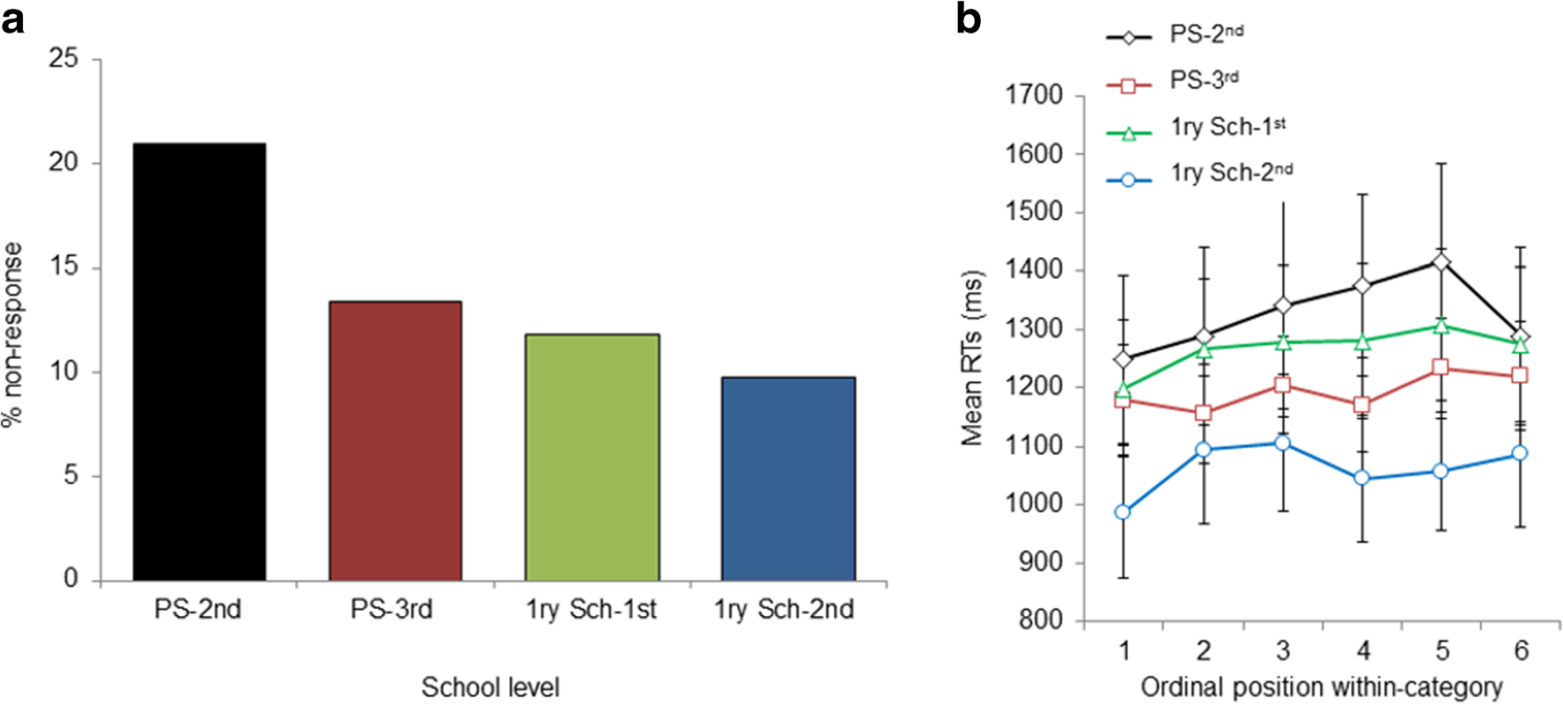
Study 2. **a** Developmental trend of nonresponses: Percentage of nonresponses in each school level. **b** Cumulative semantic costs: Mean naming latencies by ordinal position within category in each school level. As can be seen, naming latencies increase with ordinal position within category in all the school levels. All error bars are standard error of the mean. PS = preschool; 1ry Sch = primary school

The results parallel those reported in Study 1. The GLMM analysis revealed that nonresponses were more frequent in younger children, χ^2^(1) = 9.61, *p* = .001, analysis on the factor school level, χ^2^(3) = 22.53, *p* < .001. See Table 2 and Fig. 2.

The LMM analysis evaluating the developmental trend of cumulative semantic costs showed a significant main effect of trial, χ^2^(1) = 11.74, *p* < .001, and ordinal position within category, χ^2^(1) = 9.60, *p* = .001. The main effect of age, χ^2^(1) = 2.84, *p* = .091, was marginally significant. The main effect of PVVT-R was not significant, χ^2^(1) = 0.05, *p* = .809. Neither the interaction between ordinal position within category and age, χ^2^(1) = 0.19, *p* = .655, nor the interaction between ordinal position within category and Peabody, χ^2^(1) = 0.02, *p* = .885, were significant. A similar pattern of results emerged with the factor school level, the main effects of trial, χ^2^(1) = 11.74, *p* < .001, and ordinal position within category, χ^2^(1) = 9.61, *p* = .001, were significant. The main effect of school level, χ^2^(1) = 1.88, *p* = .169, was not significant. Neither the interaction between ordinal position within category and school level, χ^2^(1) = 0.019 *p* = .895, nor the interaction between ordinal position within category and Peabody, χ^2^(1) = 0.004, *p* = .945, were significant (see Appendix 4a and 4c for regression coefficients and standard errors). As can be seen in Fig. 2, RTs increased for each subsequent within-category item, while overall RTs were slower for younger children (earlier school level in Fig. 2).

In the analysis evaluating the interaction between cumulative semantic cost and nonresponse rates, it was found that RTs did not differ as a function of whether the previous within-category item was successively retrieved or was a nonresponse trial, χ^2^(1) = 1.962, *p* = .165. The effects of trial, χ^2^(1) = 5.64, *p* = .017, and ordinal position within category, χ^2^(1) = 4.61, *p* = .031, were significant. The critical interaction between previous within-category and ordinal position within-Category factors was not significant, χ^2^(1) = 0.13, *p* = .713 (see Appendix 4c for regression coefficients and standard errors). See Fig. 2.

## Discussion

In two independent picture-naming studies, we found the cumulative semantic cost effect in children. Naming latencies increased as a function of the ordinal position within category. Our results replicate, in an Italian sample, the recent cumulative semantic cost observed by Charest and Baird (2020) with native English-speaking children between 7 and 9 years of age. In addition, younger children were slower and more prone to nonresponses than were older children. This last result indicates a reduced vocabulary size in younger children, in line with previous studies (Riva et al., 2000). In the critical analysis, we explored whether the cumulative semantic cost interacts with vocabulary size and age (and school level); and whether it depends on having successfully retrieved the previous within-category item. The results indicated that vocabulary size, children’s age, and whether the previous within-category item was successfully retrieved, were not critical factors for the emergence of the phenomenon. In other words, it is the attempt to retrieve a lexical unit and not the successful retrieval of a specific lexical unit that causes cumulative semantic cost in spoken word production.

Theoretical approaches that localize cumulative semantic costs at a prelexical level of processing through an incremental weakening of the semantic-to-lexical connections (Navarrete et al., 2010; Navarrete et al., 2016; Oppenheim et al., 2010) can accommodate our results. In contrast, the competitive approach assumes that within-category words that have been named some trials before become stronger lexical competitors and hamper the retrieval of the current lexical target representation. According to this hypothesis, the cumulative semantic cost in picture naming would depend on the previously named lexical representation(s) that would exert as lexical competitor(s); by lateral inhibition in the case of Howard et al.’ (2006) model. Our findings, showing cumulative semantic cost independent of vocabulary size and vocabulary knowledge, would reject such lexical locus of the phenomenon.

Other accounts have recently been proposed to explain cumulative semantic cost in picture-naming tasks. All of them share the notion that the phenomenon is originated at the conceptual level of processing (see, for instance, Abdel Rahman & Melinger, 2009; Belke, 2013; Roelofs, 2018). These proposals may be able to accommodate our results. The present research was not designed to adjudicate between these conceptual approaches.

### Error effects in the context of semantic manipulation

While there is compelling evidence in the picture-naming task that lexical retrieval speed decreases proportionally as a function of the number of semantic pictures that have been presented before, the evidence regarding cumulative semantic cost on the probability of making an error during lexical retrieval seems to be controversial. In a picture-naming experiment, Riès et al. (2015) observed that aphasic patients were less accurate in comparison to a group of younger controls and a group of age-matched controls; however, there was no trace of cumulative semantic cost in the error rates in any of the groups. More recently, Harvey et al. (2019) have analyzed the types of errors that aphasic participants made in picture naming. These authors reported an interaction between the cumulative semantic cost and the type of errors. Specifically, semantic paraphasia errors (dog → cat) increased as a function of ordinal position within category while the rates of other types of error decreased (see, for findings in a related paradigm, Schnur et al., 2006). Error rates have been also been investigated in neurotypical (and middle age) populations. The pattern of results, however, is also incongruent, with some studies showing an effect (Navarrete et al., 2015; Navarrete et al., 2010; Rose & Abdel Rahman, 2017) and others not (Belke, 2013; Howard et al., 2006; Rose & Abdel Rahman, 2017; Runnqvist et al., 2012; Schnur, 2014; see Harvey et al., 2019, for further discussion). Finally, in the context of the child population, the only study conducted so far does not provide an analysis of the cumulative semantic cost as a function of errors type (Charest & Baird, 2020).

To offer a complete description of the results we reported here, we performed analyses on the errors of Study 2.^3^ Two analyses were performed. In the first analysis, GLMM analyses were performed on error rates. The predictors ordinal position within category and age (in months) were entered as fixed effects in the statistical model. The results showed an effect of age, χ^2^(1) = 100.72, *p* < .001, with younger children producing more errors than older children, but not a significant effect of ordinal position within category, χ^2^(1) = 0.17, *p* = .672. That is, no cumulative semantic cost was observed in the general pattern of error rates. In the second type of analysis, we distinguished between semantic paraphasias and other types of errors (e.g., circumlocutions, formal paraphasias) and explored whether these two types of errors showed cumulative effects as a function of ordinal position within category (see Harvey et al., 2019). Two GLMM analyses were performed separately, one contrasting accurate responses versus semantic paraphasias, and one contrasting accurate responses versus other types of errors. The predictor ordinal position within category was entered as a fixed effect in the two statistical models. The main effect of ordinal position within category was not significant in either of the models, neither in the model contrasting semantic paraphasias and accurate responses, χ^2^(1) = 1.526, *p* = .216, nor in the model contrasting other types of error and accurate responses, χ^2^(1) = 0.128, *p* = .720. In sum, these analyses did not report clear cumulative cost in the error rates in the child production, replicating the incongruent results in the literature with adults and aphasic populations (see Harvey et al., 2019).

### On the similarities between long-lasting semantic costs in speech production and episodic memory recall

The origin of semantic effects in speech production is generally considered within the somewhat narrow scope of language production processes; however, we believe that it may be fruitful to take a broader view, as difficulties during information retrieval induced by having previously retrieved semantically related information seems to be a broader phenomenon. For instance, a long tradition in memory research has shown that the recall of a list of words previously learned is hampered if, between the learning phase and the recall phase, participants are required to actively retrieve other exemplars of the same semantic categories, the so-called retrieval-induced forgetting (RIF; e.g., Anderson, 2003). In the classical RIF paradigm, participants study a list of category-exemplar pairs (e.g., FRUIT–banana; FRUIT–lemon; VEHICLE–car; FURNITURE–stool). In a subsequent practice phase, half of the exemplars from half of the categories are retrieved several times by presenting participants with category-letter cued stems. For instance, presented with ‘FRUIT–b’ and ‘VEHICLE–c,’ participants have to produce “banana” and “car,” respectively. In a final recall test phase, participants are asked to recall all items originally presented in the study phase. As expected, practiced items (i.e., banana, car) are recalled better than unpracticed items (i.e., lemon, stool). More surprising is that recall for unpracticed items from practiced categories (lemon) is worse than recall of unpracticed items from unpracticed categories (stool; Anderson et al., 1994; see, for evidence with children, Aslan & Bäuml, 2010).

The theoretical distinction between the weakening and the competitive accounts in the context of cumulative semantic cost in picture naming is analogous to the debate between the facilitation-based and competition-based accounts in the retrieval-induced forgetting literature (see, for discussion, Oppenheim et al., 2010). Some studies have highlighted in the past the similarities between RIF and cumulative semantic cost (Navarrete et al., 2010; Navarrete et al., 2016; Oppenheim et al., 2010), and other studies have shown empirical evidence congruent with this resemblance (Levy et al., 2007; but see Runnqvist & Costa, 2012). To this respect, the findings reported here could parallel those reported by Storm et al. (2006), who explored whether word retrieval success is a necessary condition for RIF. Under the assumption that the attempt to retrieve an item (banana) inhibits access to unpracticed items from the same category (lemon), inhibition may be independent of the successful retrieval of the practice item (banana). Storm and colleagues tested this hypothesis using a procedure in which some stem cues posed an impossible retrieval event for participants. Nonetheless, RIF was observed even in these unsuccessful conditions, suggesting that it is retrieval attempt, and not the retrieval per se, that induces inhibition on successive related retrieval attempts. The authors concluded that their findings are inconsistent with a competitive-based account of the RIF. The results we report here and the conclusion we draw from them run parallel to those of the study by Storm and colleagues in the context of RIF research and extending them to a developmental sample.

## Conclusion

In the current study, we obtained a cumulative semantic cost in children: Naming a picture hampers the subsequent lexicalization of within-category pictures in a picture-naming task. More importantly, our findings suggest that the attempt to retrieve a lexical representation and not the successful retrieval of the label per se can produce this cumulative semantic cost in spoken word production.

## **Appendix 1** Materials used in Study 1 organized by semantic category. Italian translation is provided after the English name.

Animals: dog (cane), seal (foca), hen (gallina), cat (gatto), cow (mucca), bear (orso), fish (pesce), frog (rana), snake (serpente), bird (uccello), zebra (zebra).

Body parts: ear (orecchio), heart (cuore), nose (naso), skeleton (scheletro), skull (teschio).

Buildings: church (chiesa), mill (mulino), tower (torre), bag (borsa), belt (cinta), boots (stivali), hat (cappello), jacket (giacca), sandals (sandali), shirt (camicia), skirt (gonna), vest (gilet).

Fruits: apple (mela), banana (banana), grapes (uva), lemon (limone), orange (arancia), peach (pesca), pineapple (ananas).

Furniture: bathtub (vasca), bed (letto), chair (sedia), chandelier (lampadario), desk (scrivania), sofa (divano), table (tavolo), wardrobe (armadio).

Musical instruments: drum (tamburo), guitar (chitarra), piano (pianoforte), trumpet (tromba), violin (violino).

Professionals: boxer (pugile), carpenter (falegname), cook (cuoco), fireman (pompiere), painter (pittore), singer (cantante), thief (ladro).

Tools: arrow (freccia), comb (pettine), funnel (imbuto), glass (bicchiere), hammer (martello), knife (coltello), pot (pentola), scissors (forbici).

Vehicles: ambulance (ambulanza), bike (bicicletta), carriage (carrozza), helicopter (elicottero), plane (aereo), raft (zattera), ship (nave), tractor (trattore).

## **Appendix 2** Coefficients and standard errors of the regression analysis on Study 1. **a** Regression analysis with the factor age (in months). **b** Regression analysis with the factor school level. **c** Regression analysis based on the performance on the previous within-category item (successfully naming vs. nonresponse). See main text for details.

**Table:**

(**a**)		
Fixed effects	Estimate coefficient	*SE*
Trial	1.083 × 10^-5^	3.252 × 10^-6^
Age	−7.405 × 10^-5^	6.546 × 10^-6^
Order within category	4.331 × 10^-5^	8.994 × 10^-6^
Age × Order Within Category	1.085 × 10^-6^	8.514 × 10^-6^
(**b**)		
Fixed effects	Estimate coefficient	Std. Error
Trial	1.083 × 10^-5^	3.252 × 10^-6^
School level	−8.815 × 10^-4^	7.603 × 10^-5^
Order within category	8.342 × 10^-5^	5.597 × 10^-5^
Age × Order Within Category	1.507 × 10^-5^	9.953 × 10^-6^
(**c**)		
Fixed effects	Estimate coefficient	*SE*
Trial	8.676 × 10^-6^	3.765 × 10^-6^
Previous within-category performance	4.722 × 10^-4^	5.131 × 10^-4^
Order within category	1.215 × 10^-4^	4.426 × 10^-5^
Previous Within Category Performance × Order Within Category	−8.944 × 10^-5^	1.110 × 10^-4^

## **Appendix 3** Materials used in Study 2, organized by semantic category. Italian translation is provided after the English name.

Birds: chick (pulcino), duck (anatra), eagle (aquila), parrot (pappagallo), pigeon (piccione), swan (cigno).

Body parts: ear (orecchio), eye (occhio), finger (dito), foot (piede), leg (gamba), nose (naso).

Buildings: castle (castello), church (chiesa), house (casa), mill (mulino), pyramid (piramide), tent (tenda).

Clothes: hat (cappello), shoe (scarpa), skirt (gonna), sock (calzino), trousers (pantaloni), T-shirt (maglietta).

Food: cake (torta), candy (caramella), cookie (biscotto), ice cream (gelato), lollipop (lecca-lecca), pancake (frittelle).

Fruits: apple (mela), banana (banana), lemon (limone), orange (arancia), pear (pera), strawberry (fragola).

Furniture: bed (letto), chair (sedia), sofa (divano), stool (sgabello), table (tavolo), wardrobe (armadio).

Musical instruments: accordion (fisarmonica), drum (tamburo), flute (flauto), guitar (chitarra), piano (pianoforte), trumpet (tromba).

Mammals: cat (gatto), dog (cane), horse (cavallo), pig (maiale), rabbit (coniglio), sheep (pecora).

Nature: cloud (nuvola), moon (luna), rain (pioggia), rainbow (arcobaleno), star (stella), sun (sole).

Professionals: clown (pagliaccio), cook (cuoca), doctor (dottore), fireman (pompiere), teacher (maestra), traffic warden (vigile).

Stationery: brush (pennello), felt tip pen (pennarello), pastels (pastelli), pen (penna), pencil (matita), watercolors (acquarelli).

Tableware: dish (piatto), fork (forchetta), glass (bicchiere), knife (coltello), mug (tazza), spoon (cucchiaio).

Tools: broom (scopa), bucket (secchielo), hammer (martello), saw (sega), scissors (forbici), scoop (paletta).

Vegetables: carrot (carota), eegplant (melanza), mushroom (fungo), potato (patata), tomato (pomodoro), zucchini (zucchina).

Vehicles: bicycle (bicicletta), car (macchina), moped (motorino), plane (aereo), ship (nave), train (treno).

## **Appendix 4** Coefficients and standard errors of the regression analysis on Study 2. **a** Regression analysis with the factor age (in months). **b** Regression analysis with the factor school level. **c** Regression analysis based on the performance on the previous within-category item (successfully naming vs. nonresponse). See main text for details.

**Table:**

(**a**)		
Fixed effects	Estimate coefficient	*SE*
Trial	2.203 × 10^-6^	6.428 × 10^-7^
Age	−2.092 × 10^-5^	1.426 × 10^-5^
Order within category	1.118 × 10^-4^	1.359 × 10^-4^
PVVT-R (Peabody)	1.385 × 10^-3^	9.306 × 10^-4^
Age × Order Within Category	−7.819 × 10^-7^	1.772 × 10^-6^
PVVT-R × Order Within Category	−3.959 × 10^-6^	1.140 × 10^-4^
(**b**)		
Fixed effects	Estimate coefficient	Std. Error
Trial	2.203 × 10^-6^	6.428 × 10^-7^
School level	−2.849 × 10^-4^	2.161 × 10^-4^
Order within category	6.635 × 10^-5^	7.163 × 10^-5^
PVVT-R (Peabody)	4.576 × 10^-4^	5.580 × 10^-4^
School Level × Order Within Category	−3.906 × 10^-6^	2.633 × 10^-5^
PVVT-R × Order Within Category	1.021 × 10^-5^	6.762 × 10^-5^
(**c**)		
Fixed effects	Estimate coefficient	*SE*
Trial	1.791 × 10^-6^	7.540 × 10^-7^
Previous within category performance	4.292 × 10^-5^	3.197 × 10^-4^
Order within category	4.971 × 10^-5^	2.633 × 10^-5^
Previous Within Category Performance × Order Within Category	2.770 × 10^-5^	7.534 × 10^-5^

## Footnotes

1 This phenomenon is normally called cumulative semantic interference. We use, however, the term *cumulative semantic cost* because we consider it to be theoretically more neutral.

2 We conducted a further statistical power analysis on data from Navarrete et al. (2010, Experiment 1). In that study, 20 adult participants were tested in the same experimental paradigm we used here, the continuous picture-naming task. The partial eta square for the cumulative semantic interference was 0.54. With alpha = .05 and power = 0.97, the anticipated sample size required to obtain a significant cumulative semantic interference is *n* = 14.

3 The online-accuracy coding for the normative Study 1 codified correct responses, nonresponses, and error responses without distinguishing the different types of specific errors (e.g., semantic, phonological, circumlocutions). Thus, no semantic error-type analyses could be performed for this study.
